# Same Fragments, Different Diseases: Analysis of Identical tRNA Fragments Across Diseases Utilizing Functional and Abundance-Based Databases

**DOI:** 10.3390/ncrna11050063

**Published:** 2025-08-29

**Authors:** Adesupo Adetowubo, Sathyanarayanan Vaidhyanathan, Andrey Grigoriev

**Affiliations:** 1 Department of Biology, Rutgers University, Camden, NJ 08102, USA; aaa586@scarletmail.rutgers.edu (A.A.); sv646@scarletmail.rutgers.edu (S.V.); 2 Center for Computational and Integrative Biology, Rutgers University, Camden, NJ 08102, USA

**Keywords:** tRNA-derived fragments, gene regulation, target prediction, cancer, pancreatic adenocarcinoma, gastric cancer, glioblastoma, tRF databases, Argonaute, CLASH, CLEAR, TCGA

## Abstract

**Background/Objectives:** Transfer RNA-derived fragments (tRFs) are small non-coding RNAs increasingly implicated in gene regulation and disease, yet their target specificity and disease relevance remain poorly understood. This is an exploratory study that investigates the phenomenon of identical tRF sequences reported in distinct disease contexts and evaluates the consistency between experimental findings and predictions from both target-based and abundance-based tRF databases. **Methods:** Five tRFs with identical sequences across at least two peer-reviewed disease studies were selected from a recent systematic review. Their validated targets and disease associations were extracted from the literature. Motifs and predicted targets were cross-referenced using three target-oriented databases: tatDB, tRFTar, and tsRFun. In parallel, the abundance enrichment of cancer-associated tRFs was assessed in OncotRF and MINTbase using TCGA-based abundance data. **Results:** Among the five tRFs, only LeuAAG-001-N-3p-68-85 showed complete alignment between experimental data and both tatDB and tRFTar predictions. Most of the other four displayed at least partial overlaps in motif/binding regions with some of validated targets. tRF abundance data from MINTbase and OncotRF showed inconsistent enrichment, with only AlaAGC-002-N-3p-58-75 exhibiting concordance with its experimentally validated cancer type. Most functionally relevant tRFs were not strongly represented in abundance-only databases. **Conclusions:** Given the limited number of tRFs analyzed, this study serves primarily as a pilot analysis designed to generate hypotheses and guide future in-depth research, rather than offering comprehensive conclusions. We did, however, illustrate how the analysis of tRFs can benefit from utilizing currently available databases. Target-based databases more closely reflected experimental evidence for mechanistic details when a tRF or a motif match is found. Yet all database types are incomplete, including the abundance-focused tools, which often fail to capture disease-specific regulatory roles of tRFs. These findings underscore the importance of using integrated data sources for tRF annotation. As a pilot analysis, the study provides insights into how identical tRF sequences might function differently across disease contexts, highlighting areas for further investigation while pointing out the limitations of relying on expression data alone to infer functional relevance.

## 1. Introduction

tRFs are emerging as a distinct class of small non-coding RNAs (sRNA) with potential gene regulatory functions. While tRFs have been implicated in diverse cellular processes, including modulation of translation, apoptosis, and immune signaling, our understanding of their molecular targets and mechanisms of action remains limited. Similarly to microRNA (miRNA), tRF roles in posttranscriptional gene regulation involving interactions with mRNA targets were proposed [1,2]. A significant bottleneck in this field is the scarcity of experimentally validated tRF–target interactions. Most studies focus on one or two tRFs in isolation, often lacking functional assays to confirm predicted targets, which limits generalizability and mechanistic insight [3]. Amid this knowledge gap, numerous small RNA sequencing efforts have cataloged large numbers of tRFs, many differing by just one nucleotide. Databases like MINTbase [4] list many thousands of human tRFs based on abundance profiles, but typically without accompanying target predictions or mechanistic evidence.

In the past decade, specialized resources have emerged to support target discovery based on large-scale experimental screens capturing sRNA–target pairs. The CLASH (Crosslinking, Ligation, and Sequencing of Hybrids) protocol was initially designed to capture miRNA–target interactions associated with Argonaute (AGO) proteins [5]. Subsequently, it was discovered that the dataset also included tRFs and their target interactions [1,6]. miRNA regulates its targets through base pairing with its seed region at the 5′ end. Similarly, many tRFs possess miRNA-like seed regions, enabling them to engage with specific targets, including protein-coding genes [1,2,7]. The CLEAR (Covalent Ligation of Endogenous Argonaute-bound RNAs)-CLIP experiments enhanced standard CLIP methods by elucidating the connections between RNA and RNA-binding proteins, such as AGO, while also ligating the RNA–target interaction. This procedure incorporates crosslinking, ligation of the sRNA–target interaction, and subsequent immunoprecipitation of the complex, followed by sequencing [8]. This approach is conceptually similar to the CLASH protocol and aims to reveal sRNA–target interactions.

Data from these large-scale experiments have been analyzed and placed in online databases, and here we incorporate results from two of those. tatDB integrates CLASH data to provide transcriptome-wide evidence of Argonaute-mediated tRF–mRNA interactions, along with binding motifs and hybrid structures [9]. tRFTar offers complementary predictions based on machine learning models trained on CLASH and CLEAR-CLIP data [10]. Both CLASH and CLEAR-CLIP produce large numbers of false positives (e.g., tRF–target pairs not loaded to AGO) and negatives (e.g., pairs missing in HEK293 cells) and require careful bioinformatics analyses before placing them in a database. Further, finding common sequence motifs among the divergent targets of the same tRF, supported by a threshold of minimum free energy of interaction of such motifs with a putative tRF seed region, can provide additional evidence of tRF binding to its targets.

These databases store identified motif patterns to predict potential binding sites of tRFs with their respective targets. Both databases are used here as tools for motif matching and target verification, though limitations related to experimental context and prediction bias remain.

In contrast, resources such as the OncotRF database catalog cancer-associated tRFs based on abundance data rather than target predictions. OncotRF integrates small RNA and mRNA abundance profiles across The Cancer Genome Atlas (TCGA) to assess the cancer relevance of tRFs, including tumor–normal abundance comparisons and survival analyses [11]. Similarly, MINTbase v2.0 compiles the abundance of tRFs across normal and cancerous tissues, enabling disease enrichment analysis without incorporating target predictions [4]. These resources offer valuable context for understanding abundance dynamics, but lack the ability to confirm functional targeting, making them complementary to tatDB and tRFTar in evaluating tRF roles in disease.

In a recent comprehensive literature review, over 100 studies were analyzed to map the involvement of tRFs across disease contexts [3]. The review identified several cases in which identical tRF sequences were independently implicated in multiple, unrelated disease studies. This observation raises fundamental questions about the reuse of certain tRFs in regulatory processes. Do such phenomena occur through shared molecular mechanisms, commonalities of disease pathways, or targeting unrelated mRNAs?

Here, we directly extend the findings of the previous review, concentrating on such lesser-known phenomenon of identical tRFs being detected in different pathological conditions. The objectives are threefold:

(1) To catalog and compare instances of repeated tRFs associated with diseases;
(2) To check the consistency of reported targets through literature- and database-supported motif verification to investigate if these overlaps signify biologically significant convergence or merely incidental sequence reuse;
(3) To assess whether enrichment patterns from abundance-based databases align with disease-specific roles inferred from experimental validation in disease contexts.

This research aims to enhance our understanding of the sequence-specific functions of tRFs and to pinpoint promising candidates for future experimental validation. It also illustrates the value that can be extracted from comparative literature analyses and database queries to advance our understanding of how tRFs may target mRNAs. It is important to note that this investigation, which focuses on a small subset (*n* = 5) of identical tRFs across distinct diseases, should be viewed as an exploratory pilot analysis. The findings presented are limited in scope and intended to generate hypotheses for more comprehensive future studies.

## 2. Results

The naming convention adopted in this study primarily follows the standardized tRF IDs provided by tatDB, which are based on genomic origin, strand, and position of the tRF within the parent tRNA [9]. For all five selected tRFs, unique tatDB identifiers were assigned based on full sequence matches.

### 2.1. Candidate tRFs

Five tRFs were selected based on exact sequence identity across at least two peer-reviewed studies from distinct disease contexts identified in a recent literature review [3]. These included GlyGCC-001-N-5i-1-33, GlyCCC-001-N-5i-1-32, GluCTC-001-N-5p-1-31, LeuAAG-001-N-3p-68-85, and AlaAGC-002-N-3p-58-75 (Table 1). These fragments were independently implicated in pairs of diverse conditions, including gastric cancer and glioblastoma [12,13], Alzheimer’s disease and ischemia [14,15], Huntington’s disease and atherosclerosis [16,17], and pancreatic cancer and ischemic stroke [18,19].

### 2.2. Data Extraction and Annotation

Each tRF was annotated with its complete nucleotide sequence, disease associations, experimentally validated targets, and seed or functional motifs as reported in the original literature. GlyGCC-001-N-5i-1-33 was shown to inhibit *STAT3* in gastric cancer [13]. GlyCCC-001-N-5i-1-32 was shown to be upregulated in response to ischemia and experimentally validated to inhibit angiogenesis by impairing endothelial cell proliferation, migration, and tube formation. Its biogenesis was linked to angiogenin (ANG)-mediated cleavage under hypoxic conditions [14]. The same tRF was reported in Alzheimer’s disease without direct target validation [15]. GluCTC-001-N-5p-1-31 was reported to regulate *SRF* and *ARRB* in neurological and vascular disease models [16,17]. LeuAAG-001-N-3p-68-85 was validated to target *UPF1*, a regulator of mRNA decay [19], while AlaAGC-002-N-3p-58-75 was linked to the suppression of *ASCL2*, a transcription factor involved in cancer progression [18]. The validated targets and associated disease phenotypes are summarized in Table 1, together with the respective tRFs.

### 2.3. Database Cross-Referencing of tRF Sequences

Cross-referencing each complete tRF sequence and its associated seed motifs was performed using tatDB, tRFTar, and tsRFun. tsRFun returned no relevant predictions for any of the selected tRFs and was excluded from further analyses. For all five tRFs, we observed either a full sequence match or partial seed region overlaps between experimentally reported motifs and those predicted in tatDB or tRFTar.

For GlyGCC-001-N-5i-1-33, which had a validated target of *STAT3* with the reported seed CATGGG [13], tatDB returned a similar motif ATGGGTGG, while tRFTar returned a longer ATGGGTGGTTCAGTGGTAG. Neither database listed *STAT3* as a predicted target; however, a highly similar motif suggests potential binding, undetected by the CLASH protocol in the HEK293 cell line. tatDB identified *EIF2AK1* as a target for the complete tRF sequence, while tRFs with a single nucleotide extension at the 3′ end were predicted to target *EEF1A1*, *FASN*, and *GAPDH* genes involved in stress response, protein translation, lipid metabolism, and glycolysis (Table 2). This suggests additional regulatory potential for the core motif sequence across different pathways.

GlyCCC-001-N-5i-1-32, was linked to ANG-mediated cleavage under ischemic conditions, without a specific binding region [14]. When queried against tatDB, it returned a longer variant of the tRF sequence with a CCCAC extension with Neogenin 1 (*NEO1*) as a predicted target and CTCGCCTCCCAC as the seed region (Table 2).

GluCTC-001-N-5p-1-31was reported to regulate *SRF* and *ARRB* via a binding region CCCTGG [16,17]. In tatDB and tRFTar, *SRF* and *ARRB* were not listed among direct predictions. Interestingly, further analyses revealed both *SRF* and *ARRB* were listed by tatDB as targets of GluCTC-001-N-5p-1-33, which is two nucleotides (nt) longer. Further, GluCTC-001-N-5p-1-20, which is 11 nt shorter, targeted *SRF*, and GluCTC-001-N-5p-1-22, 9 nt shorter, targeted *ARRB*. All of these tRFs arise from the same tRNA and have the same motif TCCCTGGTGGTC, a superstring of CCCTGG, collectively indicating possible binding of this region with both *SRF* and *ARRB*. Another tRF, GluTTC-001-N-5p-1-19, also has this motif and targets *SRF*.

LeuAAG-001-N-3p-68-85 showed full agreement between the database predictions and the sequence/motif in the original experimental paper [19], with *UPF1* as a validated target in both tatDB and tRFTar. In contrast, AlaAGC-002-N-3p-58-75 demonstrated partial motif overlap in both databases, but *ASCL2* did not appear in any target predictions.

To further investigate disease-specific associations, all five tRFs were queried in the OncotRF database. Only two, LeuAAG-001-N-3p-68-85 and AlaAGC-002-N-3p-58-75, were returned with detectable abundance in tumor samples. These tRFs appeared in datasets for cancers such as bladder carcinoma (BLCA), breast cancer (BRCA), and esophageal carcinoma (ESCA), though no explicit functional annotation or target association was provided. The remaining four tRFs, including those with experimentally validated roles in tumor progression (e.g., GlyGCC-001-N-5i-1-33 targeting *STAT3*), were absent from OncotRF results. This limited overlap suggests that OncotRF, while responsive, shows poor agreement with functional tRF–disease relationships found in focused experiments.

### 2.4. Cross-Referencing of Target Genes

**Figure 1:**
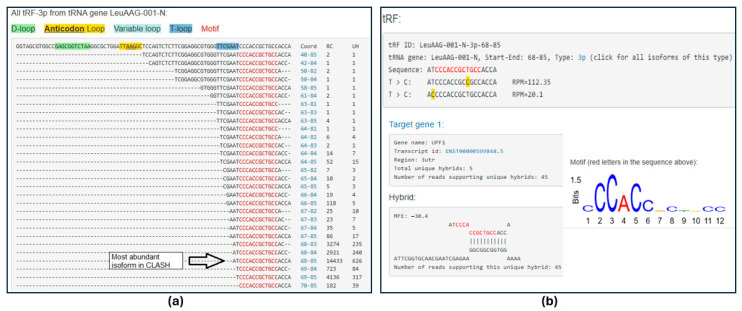
(**a**) A snapshot of a results page from tatDB, showcasing all tRF-3p derived from the tRNA gene LeuAAG-001-N, as identified from the CLASH data. The tRNA sequence is on top; colors show various tRNA regions, including the identified motifs. Each tRF is shown with its positional coordinates (Coord) relative to the tRNA gene, observed read counts (RC), and unique hybrids (UH). (**b**) tatDB details for the most abundant tRF isoform in (**a**), with the motif marked in red. Below the tRF, the T > C conversion sites from PAR-CLIP data are highlighted in yellow with their frequency (RPM). Such conversion sites typically occur outside the binding regions, helping one to home in on the latter. The lower panel shows a predicted RNA–RNA hybrid structure generated by RNAhybrid, indicating binding between the tRF and its experimentally validated target *UPF1*. The observed motif is also shown as a sequence logo on the right.

To complement the sequence-based searches, each experimentally validated target gene was queried independently in all three databases. *UPF1* returned exact or near-exact sequence matches in both tatDB and tRFTar, strongly supporting its experimentally validated targeting by LeuAAG-001-N-3p-68-85. Figure 1 from tatDB illustrates such database support with the frequent occurrence of this isoform in sequencing read counts and unique hybrids. Motif presence in related isoforms, as calculated by MEME [21], additionally indicates a statistically significant overrepresentation of complementary sequences, reinforcing this validation.

**Figure 2:**
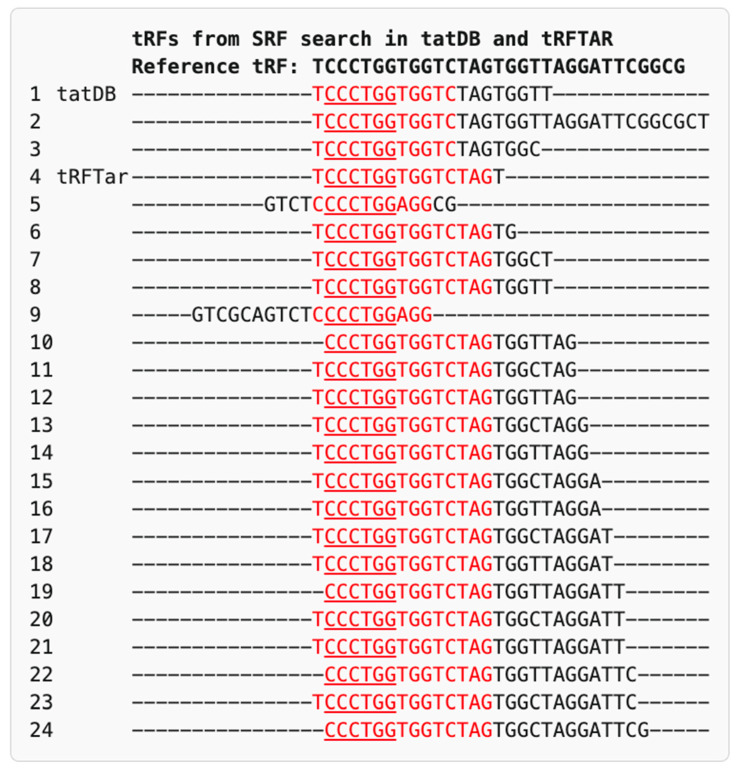
Alignment of tRF sequences from tatDB and tRFTar databases predicted target *SRF*. The reference tRF (GluCTC-001-N-5p-1-31; sequence: TCCCTGGTGGTCTAGTGGTTAGGATTCGGCG) is aligned with the predicted tRFs retrieved from the tatDB and tRFTar databases. Red font highlights overlapping motifs with the experimentally validated seed region (CCCTGG, underlined).

**Figure 3:**
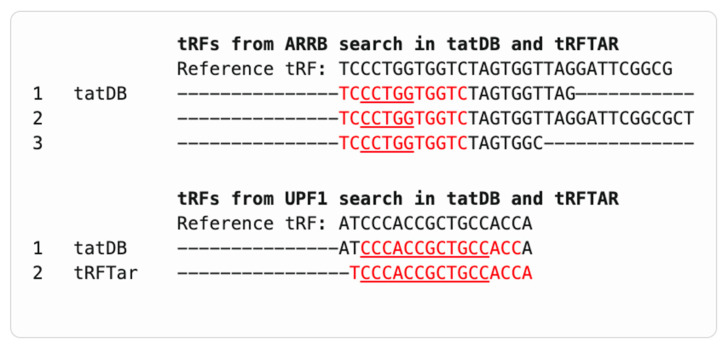
Alignment of tRF sequences from tatDB and tRFTar databases predicted targets *ARRB* and *UPF1*. The reference tRF sequences (GluCTC-001-N-5p-1-31 for *ARRB*: TCCCTGGTGGTCTAGTGGTTAGGATTCGGCG, and LeuAAG-001-N-3p-68-85 for *UPF1*: ATCCCACCGCTGCCACCA) are aligned with predicted tRFs retrieved from tatDB and tRFTar. Red font indicates overlapping motifs with experimentally validated seed regions (*ARRB*: CCCTGG; *UPF1*: CCCACCGCTGCC, underlined).

*STAT3* returned 9 hits in tatDB and 57 in tRFTar, though none contained the CATGGG motif. *SRF* and *ARRB* showed partial motif overlap in tatDB and tRFTar; three hits in tatDB and twenty in tRFTar shared the core CCCTGG sequence (Figure 2 and Figure 3, following the display principle of Figure 1a). For completeness, the *UPF1* near-exact sequence match in tRFTar, corresponding to truncated LeuAAG-001-N-3p-68-85, is shown (Figure 3). *ASCL2* returned no hits in any database.

### 2.5. Abundance Data on tRFs in OncotRF and MINTbase

To investigate whether tRF abundance in tumor samples supports the disease associations reported in the literature, three cancer-implicated tRFs were queried in both MINTbase and OncotRF.

GlyGCC-001-N-5i-1-33, validated as targeting *STAT3* in gastric cancer [13], was not detected in OncotRF and showed inconsistent or low abundance in MINTbase, with evident variability across non-TCGA datasets and TCGA dataset abundance near the baseline (although this may be an artifact of the MINTbase display).

LeuAAG-001-N-3p-68-85, which targets *UPF1* in pancreatic cancer, was shown in the original study to be significantly upregulated in pancreatic cancer cell lines (BxPC-3 and PANC-1) relative to normal pancreatic duct epithelial cells (HPDE6c7), with relative abundance levels exceeding 3.5-fold [19]. Data on this tRF from OncotRF revealed a more modest upregulation in pancreatic adenocarcinoma (PAAD), with a median tumor.

**Figure 4:**
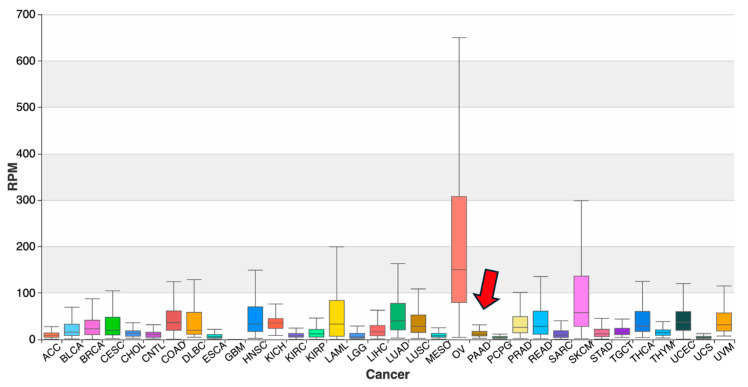
Abundance distribution of LeuAAG-001-N-3p-68-85 across TCGA cancers reported by OncotRF. Boxplot showing RPM values for LeuAAG-001-N-3p-68-85 across tumor types in TCGA datasets. Highest median abundance was observed in ovarian cancer (OV), followed by endometrial carcinoma (UCEC) and skin cutaneous melanoma (SKCM), while PAAD (pancreatic adenocarcinoma, arrow), where this tRF has experimentally validated targets, showed moderate levels. Disease abbreviations can be found in Table A1 in Appendix A.

It also had an RPM of 11.29 compared to 5.42 in normal tissue, and much higher levels observed in cancers such as ovarian (OV; median RPM of ~150, Figure 4) and skin cutaneous melanoma (SKCM; median RPM of ~50). Similarly, MINTbase showed widespread detectability of the tRF across TCGA cancers but the ranges of enrichment in PAAD were much lower than controls (with OV and SKCM again showing the highest enrichment relative to all cancer types), in contrast with the pancreatic cancer cell lines [19].

**Figure 5:**
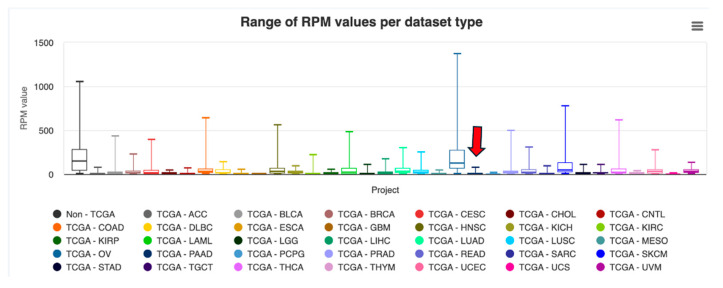
Range of absolute RPM values across TCGA projects for LeuAAG-001-N-3p-68-85, as reported by MINTbase. The red arrow indicates the dataset corresponding to PAAD. Disease abbreviations can be found in Table A1 in Appendix A.

AlaAGC-002-N-3p-58-75 displayed a modest yet consistent abundance trend in PAAD across both MINTbase and OncotRF, but these levels did not quite match the upregulation (3.5 in tumors versus 1.5 in normal) observed in the original publication [18]. In OncotRF, median RPM in PAAD tumor samples was 20.38 compared to 14.475 in normal samples, and 179 out of 183 tumor samples showed detectable levels (RPM > 1), indicating a modest differential abundance. In MINTbase, AlaAGC-002-N-3p-58-75 was broadly abundant across TCGA cancers, with PAAD falling within the moderate abundance range, but without specific enrichment relative to other tumor types such as OV or SKCM (Figure 5). These discrepancies highlight the limitation of relying solely on abundance-based databases to assess the functional and disease relevance of tRFs.

## 3. Discussion

This study highlights an intriguing yet underexplored aspect of tRF research, the recurrence of identical tRF sequences across diverse disease contexts. Initially, it was hypothesized that identical tRF sequences might imply conserved or consistent regulatory interactions across distinct pathological conditions [3]. However, further analysis revealed significant variability, suggesting predominantly context-dependent functional roles rather than universal targeting patterns.

Cross-referencing experimental findings with data from tatDB and tRFTar databases yielded mixed results. Database predictions based on large-scale experiments provided valuable but partial support, notably, a clear agreement on the experimentally confirmed target, *UPF1* [19], for LeuAAG-001-N-3p-68-85. This represents an important instance where experimental and computational (or small-scale and large-scale) datasets strongly align. In contrast, other tRFs—including GlyGCC-001-N-5i-1-33, GlyCCC-001-N-5i-1-32, and GluCTC-001-N-5p-1-31—lacked direct database predictions matching experimentally validated targets such as *STAT3*, *SRF*, and *ARRB.* These findings underscore the inherent limitations in the coverage of tRF–target space by CLASH and CLEAR studies and the resulting computational prediction models, likely attributable to methodological biases or an incomplete understanding of tRF targeting mechanisms. In light of this limited coverage, the partial motif overlaps for tRFs targeting *SRF* and *ARRB* described here serve as valuable indirect support for putative binding regions.

Several reasons could explain discrepancies between computational predictions from databases like tatDB and tRFTar and experimentally validated targets. First, these predictions often rely heavily on large-scale datasets such as CLASH, derived predominantly from HEK293 cells, which may not reflect tissue-specific interactions. Second, tRF isoform variability (due to biogenesis pathways or post-transcriptional modifications of the tRNA of origin) may alter their binding affinity and target specificity, complicating computational predictions [21]. Modifications such as methylation or pseudouridylation of tRFs might significantly affect RNA–RNA interactions but remain largely unaccounted for in current predictive models. Integration of such data could improve the reliability of target identification and/or tRF ability to bind them in different conditions. Other future improvements could leverage tissue-specific AGO-CLIP datasets to enhance the accuracy of computational predictions.

The paucity of relevant matches in tsRFun further underscores the need for comprehensive data integration and more rigorous experimental validation methodologies. Consistent database maintenance may elevate the relevance of other databases that were unavailable during the testing and writing of this paper.

In contrast to target-based databases, abundance-only resources like OncotRF and MINTbase provided similarly limited insight into functional relevance and limited agreement with the results of smaller-scale studies. Among the three cancer-implicated tRFs queried, GlyGCC-001-N-5i-1-33 (validated in gastric cancer) was absent from OncotRF and showed inconsistent representation in MINTbase, with weak or variable abundance across TCGA-STAD datasets. LeuAAG-001-N-3p-68-85 was widely detectable but showed modest abundance in pancreatic cancer, its experimentally validated context, and much stronger signals in unrelated cancers like ovarian (OV) cancer and melanoma (SKCM), prompting one to exercise caution in interpreting TCGA sRNA results.

However, these discrepancies may also indicate the potential functional and targeting significance of LeuAAG-001-N-3p-68-85 in OV and SKCM. Moreover, a weak enrichment in the TCGA-STAD, despite functional relevance demonstrated in gastric cancer, may suggest that LeuAAG-001-N-3p-68-85 also exerts its regulatory function at relatively low abundance levels or in a cell-type specific manner not fully captured by bulk tissue RNA-seq datasets. On the other hand, AlaAGC-002-N-3p-58-75 demonstrated high abundance in PAAD and other tumors across both platforms, providing one of the few cases where abundance enrichment aligned with functional data [18]. These patterns reinforce the view that, while abundance-based resources can support the contextual relevance of certain tRFs, they often fail to capture functional specificity observed in focused small-scale studies.

In other words, while abundance-based databases such as OncotRF and MINTbase can provide insight into general tRF detectability across cancers, they do not consistently align with experimentally validated disease associations or reflect the magnitude of abundance observed in functional studies. While this could be expected, our results indicate that such abundance results should be interpreted with caution. Target-based databases (tatDB, tRFTar) serve a different (more mechanistic) purpose and reliably capture validated tRF–target interactions in some instances (such as LeuAAG-001-N-3p-68-85 targeting *UPF1*) but fail to predict several experimentally confirmed targets for other tRFs. This emphasizes the importance of target-based resources in mechanistic studies while highlighting the necessity for ongoing improvement of both abundance- and target-based platforms and a need for integrating these.

Several previously developed databases seem unmaintained or offline, further complicating cross-referencing and comparative analyses. This instability emphasizes the pressing requirement for consistently curated and experimentally enriched resources. Expanding these resources to incorporate multidimensional data such as cellular localization, RNA secondary structures, tissue-specific RNA-binding proteins, and functional assay outcomes would significantly enhance predictive accuracy and biological inference capabilities.

This study’s limitations, consistent with its exploratory nature, include reliance on the existing literature for candidate selection, which may bias the results toward particular diseases or tRFs that are more commonly studied. It is important to recognize that the databases primarily include data from large-scale studies performed on HEK293 cells, and we compare these with results of small-scale experiments aimed at exploring specific questions about tRF roles in various disease contexts. We note that the functions and potential roles of tRFs may vary between different cells, and differences in experimental validation techniques may hinder direct functional comparisons.

To tackle these challenges in upcoming research, it is essential to implement standardized and rigorous experimental testing in various contexts. By integrating computational methods with broader experimental validation, future research could elucidate whether the identical tRF sequences genuinely indicate biological convergence and common features of targets or if they serve context-dependent regulatory functions.

Ultimately, our findings advocate for a cautious interpretation of tRF–target predictions based solely on sequence identity or large-scale sRNA-seq datasets. We stress the use of integrated, experimentally benchmarked computational tools and cross-checked data sources. Such holistic approaches will be vital for unraveling the complex biology underlying tRF function and exploiting their potential in diagnostics and therapeutics across distinct pathological conditions.

## 4. Materials and Methods

### 4.1. Selection of Candidate tRFs

Candidate tRFs were identified from a previously published systematic review of over 100 peer-reviewed studies on tRF involvement across disease contexts [3]. To select the tRFs for analysis, we applied the following criteria:

- The tRF sequence had to be exactly identical (full sequence match) across at least two independently published studies.
- Each matched tRF sequence had to be associated with distinct disease contexts, indicating its potential multifunctional or pleiotropic role across diseases.
- Studies reporting functional overlaps with divergent (non-identical) tRF sequences were explicitly excluded to ensure a clear and unambiguous focus on exact sequence matches and their implications.

Following these criteria, a total of five tRF sequences were selected for inclusion in this pilot analysis. A complete list of all tRF sequences initially considered is available in our previously published review [3].

### 4.2. Data Extraction and Annotation

For each tRF pair, the information listed was manually extracted from the original publications (Table 3).

### 4.3. Database Cross-Referencing of tRF Sequences

To assess the consistency of reported interactions and support motif verification, each tRF sequence and seed region was queried against the databases in Table 4.

These databases were accessed in May 2025 and used in combination to provide both abundance-based context and motif-level targeting potential. tRFs with known experimental evidence were cross-referenced with entries in these repositories to evaluate consistency and possible abundance-level enrichment across disease types. The presence of identical or similar motifs in other tRFs targeting the same gene was flagged as indirect evidence supporting regulatory potential.

It is important to note that several other databases previously published for tRF research, such as early tsRBase, were found to be offline or nonfunctional at the time of this analysis and, therefore, were excluded.

### 4.4. Cross-Referencing of Target Genes and Motif-Level Confirmation

To assess whether the target genes of the identical tRFs reported in the literature might also be targeted by other tRFs through shared motifs, each validated target gene (e.g., *STAT3*, *SRF*, *UPF1*) was queried separately in the three databases above.

The objective was to determine whether any other tRFs within these databases, possibly including the tRFs in question, contained matching seed regions or motifs that targeted the same gene. Motif overlaps were recorded and compared with those from experimental papers.

Motifs reported in the original papers for each tRF (when available) were compared with motifs stored in the databases above. Seed region overlaps were used to verify whether the paper-reported tRFs had consistent targeting patterns with known entries in these databases. Motif overlap with other tRFs was considered potential evidence of convergent targeting behavior.

## Figures and Tables

**Table 1:** Information about the five selected tRFs, their targets, and their roles in their respective diseases. Each tRF is identified by its tatDB-based ID, nucleotide sequence, and disease contexts. Validated targets refer to experimentally confirmed gene interactions reported in original studies. Abbreviations: *STAT3*—signal transducer and activator of transcription 3; *ANG*—angiogenin; *SRF*—serum response factor; *ARRB*—arrestin beta-1; *UPF1*—regulator of nonsense-mediated mRNA decay; *ASCL2*—achaete-scute family bHLH transcription factor 2.

tRF ID	Sequence (5′–3′)	Disease Contexts	Validated Target(s)	Reported Functional Role
GlyGCC-001-N-5i-1-33	GCATGGGTGGTTCAGTGGTAGAATTCTCGCCTG	Gastric cancer [13]; Glioblastoma [12]	*STAT3* None	Suppression of tumor progression via *STAT3* inhibition
GlyCCC-001-N-5i-1-32	GCATTGGTGGTTCAGTGGTAGAATTCTCGCCT	Alzheimer’s disease [15]; Ischemia [14]	None	Inhibition of angiogenesis via modulation of endothelial cell function; biogenesis linked to angiogenin activity
GluCTC-001-N-5p-1-31	TCCCTGGTGGTCTAGTGGTTAGGATTCGGCG	Huntington’s disease [17]; Atherosclerosis [16]	*SRF*; *ARRB*	Regulation of neuronal and vascular inflammation pathways
LeuAAG-001-N-3p-68-85	ATCCCACCGCTGCCACCA	Pancreatic cancer [19]; Stroke [20]	*UPF1*; None	Promotion of tumor proliferation via *UPF1* suppression
AlaAGC-002-N-3p-58-75	TCCCCGGCACCTCCACCA	Pancreatic adenocarcinoma [18]; Stroke [20]	*ASCL2* None	Promotion of tumor proliferation via *ASCL2* inhibition

**Table 2:** tatDB-predicted gene targets of GlyGCC-001-N-5i-1-33 and GlyCCC-001-N-5i-1-32 and their closely related tRF variants. Targets were identified from tatDB by querying the exact or extended guide sequences. Seed region is highlighted in red.

Guide Sequence (5′–3′)	Target Gene
GCATGGGTGGTTCAGTGGTAGAATTCTCGCCTG	*EIF2AK1*
GCATGGGTGGTTCAGTGGTAGAATTCTCGCCTGC	*EEF1A1*
GCATGGGTGGTTCAGTGGTAGAATTCTCGCCTGC	*FASN*
GCATGGGTGGTTCAGTGGTAGAATTCTCGCCTGC	*GAPDH*
GCATTGGTGGTTCAGTGGTAGAATTCTCGCCTCCCAC	*NEO1*

**Table 3:** Data types extracted from original publications for each tRF along with their description.

Data Type	Description
tRF Nucleotide Sequence	Full tRF sequence (5′–3′) extracted from original publications.
Disease Context	Disease(s) in which each tRF was reported to play a role.
Experimentally Validated Targets	Genes confirmed as targets using qRT-PCR, luciferase reporter assays, or Western blot.
Seed Region or Motif	Reported functional motifs or seed regions relevant to target interaction.
Validation Metadata	Position of interaction and evidence tier (e.g., high-confidence vs. putative).

**Table 4:** Databases utilized for acquiring information on tRFs, targets, or motifs.

Database	Purpose
tatDB [9]	Provided CLASH-based evidence for tRF–mRNA interactions, motif matches, and hybrid structures in AGO1-loaded complexes.
tRFTar [10]	Offered computational target predictions using machine learning trained on CLASH and CLEAR-CLIP datasets.
tsRFun [22]	Enabled functional enrichment analysis of tRFs and associated targets in human tissues.
OncotRF [11]	Displayed tumor–normal abundance data (RPM) across TCGA datasets; no target predictions included.
MINTbase v2.0 [4]	Visualized tRF distribution across TCGA and non-TCGA projects using sequence queries and abundance filters (e.g., RPM ≥ 1).

## Data Availability

The original contributions presented in this study are included in the article. Further inquiries can be directed to the corresponding author.

## Appendix A

**Table A1:** Full names and abbreviations of cancer types from The Cancer Genome Atlas (TCGA) referenced in Figure 4 and Figure 5.

Abbreviation	Cancer Type (Full Name)
ACC	Adrenocortical Carcinoma
BLCA	Bladder Urothelial Carcinoma
BRCA	Breast Invasive Carcinoma
CESC	Cervical Squamous Cell Carcinoma and Endocervical Adenocarcinoma
CHOL	Cholangiocarcinoma
COAD	Colon Adenocarcinoma
DLBC	Diffuse Large B-cell Lymphoma
ESCA	Esophageal Carcinoma
GBM	Glioblastoma Multiforme
HNSC	Head and Neck Squamous Cell Carcinoma
KICH	Kidney Chromophobe
KIRC	Kidney Renal Clear Cell Carcinoma
KIRP	Kidney Renal Papillary Cell Carcinoma
LAML	Acute Myeloid Leukemia
LGG	Lower Grade Glioma
LIHC	Liver Hepatocellular Carcinoma
LUAD	Lung Adenocarcinoma
LUSC	Lung Squamous Cell Carcinoma
MESO	Mesothelioma
OV	Ovarian Serous Cystadenocarcinoma
PAAD	Pancreatic Adenocarcinoma
PCPG	Pheochromocytoma and Paraganglioma
PRAD	Prostate Adenocarcinoma
READ	Rectum Adenocarcinoma
SARC	Sarcoma
SKCM	Skin Cutaneous Melanoma
STAD	Stomach Adenocarcinoma
TGCT	Testicular Germ Cell Tumors
THCA	Thyroid Carcinoma
THYM	Thymoma
UCEC	Uterine Corpus Endometrial Carcinoma
UCS	Uterine Carcinosarcoma
UVM	Uveal Melanoma
CNTL	Control (non-cancer samples)
Non-TCGA	Non-TCGA dataset
